# Survivin enhances hippocampal neurogenesis and cognitive function in Alzheimer's disease mouse model

**DOI:** 10.1111/cns.14509

**Published:** 2023-10-30

**Authors:** Yeongae Lee, Yeon‐Joo Ju, Min Sung Gee, Seung Ho Jeon, Namkwon Kim, Taeyoung Koo, Jong Kil Lee

**Affiliations:** ^1^ College of Pharmacy Kyung Hee University Seoul Korea

**Keywords:** 5XFAD mouse, Alzheimer's disease, neurogenesis, proneural genes, survivin

## Abstract

**Aims:**

Cognitive impairment is associated with reduced hippocampal neurogenesis; however, the causes of decreased hippocampal neurogenesis remain highly controversial. Here, we investigated the role of survivin in the modulation of hippocampal neurogenesis in AD.

**Methods:**

To investigate the effect of survivin on neurogenesis in neural stem cells (NSCs), we treated mouse embryonic NSCs with a survivin inhibitor (YM155) and adeno‐associated viral survivin (AAV‐Survivin). To explore the potential role of survivin expression in AD, AAV9‐Survivin or AAV9‐GFP were injected into the dentate gyrus (DG) of hippocampus of 7‐month‐old wild‐type and 5XFAD mice. Cognitive function was measured by the Y maze and Morris water maze. Neurogenesis was investigated by BrdU staining, immature, and mature neuron markers.

**Results:**

Our results indicate that suppression of survivin expression resulted in decreased neurogenesis. Conversely, overexpression of survivin using AAV‐Survivin restored neurogenesis in NSCs that had been suppressed by YM155 treatment. Furthermore, the expression level of survivin decreased in the 9‐month‐old 5XFAD compared with that in wild‐type mice. AAV‐Survivin‐mediated overexpression of survivin in the DG in 5XFAD mice enhanced neurogenesis and cognitive function.

**Conclusion:**

Hippocampal neurogenesis can be enhanced by survivin overexpression, suggesting that survivin could serve as a promising therapeutic target for the treatment of AD.

## GA

Survivin is able to regulate neurogenesis in vitro and in vivo model. Our results demonstrate that therapeutic effects of survivin which improves cognitive function by enhancing neurogenesis in 5XFAD mouse. Survivin seems to be a good candidate for therapeutics to restore cognitive function in hippocampus of AD.

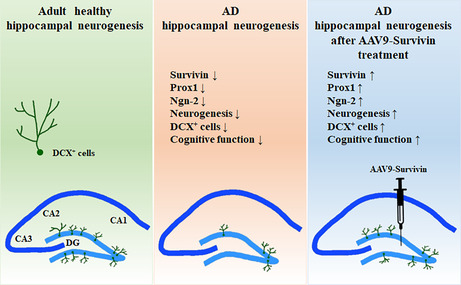

## 1 INTRODUCTION

Alzheimer's disease (AD) is a neurodegenerative disease characterized by memory loss and progressive cognitive decline.^1^ In addition, the neuropathological symptoms of AD are attributed to the accumulation of insoluble amyloid β fibers on the outside and tau protein on the inside of the neurons.^2^, ^3^ During this process, the neurons in the hippocampus and cerebral cortex are damaged, leading to neuronal cell loss.

Adult neurogenesis is the generation of new neurons from neural stem cells (NSCs) in the dentate gyrus (DG) and subventricular zone (SVZ).^4^, ^5^ Neurogenesis declines with aging, and impairment of hippocampal neurogenesis is linked to cognitive decline.^6^, ^7^ Recent studies have reported reduced neurogenesis in the hippocampus of AD mouse models and early stage of AD patients.^6^, ^7^, ^8^, ^9^ Conversely, increased neurogenesis in AD animal models has been shown to restore cognitive function.^8^, ^10^, ^11^, ^12^ Therefore, the regulation of hippocampal neurogenesis is crucial for the prevention and treatment of AD. However, further research is required to understand the mechanisms underlying the regulation of neurogenesis and the reasons for this decline in AD.

Survivin belongs to the inhibitor of apoptosis protein (IAP) family, which plays a role in apoptosis inhibition and cell cycle regulation.^13^ While most studies on survivin have focused on its involvement in cancer,^14^, ^15^, ^16^ research has also demonstrated its significance in brain function, particularly in neurogenesis and the early development of the central nervous system.^17^, ^18^ In addition, a decrease in neurogenesis was also observed when the expression of survivin was reduced due to the decrease of Wnt,^19^ and survivin regulates Prospero‐related homeodomain transcription factor 1 (Prox1), which is one of the transcription factors to enhance the Wnt/β‐Catenin pathway.^20^, ^21^ Prox1 is expressed in neuroblasts, immature neurons, and mature neurons, and it induces differentiation into neurons.^21^, ^22^, ^23^ Also downregulation of survivin correlates with alterations in adult hippocampal neurogenesis and apoptosis, resulting in impaired spatial learning and memory following traumatic brain injury.^24^ Through the following research results, survivin seems to play an important role in neurogenesis and cognitive ability.

Accumulating evidence suggests a potential association between survivin levels, neurogenesis, and cognitive function. However, the relationship between survivin and neurogenesis in AD remains unclear. Therefore, the objective of this study was to investigate survivin modulation of hippocampal neurogenesis in the AD mouse model. We employed both in vitro and in vivo experimental systems to evaluate the effect of survivin on AD‐related neurogenesis.

## 2 MATERIALS AND METHODS

### 2.1 Construction of an AAV vector plasmid encoding survivin

A mouse codon‐optimized survivin coding sequence was synthesized and the sequence was cloned into an adeno‐associated virus (AAV) inverted terminal repeat (ITR)‐based vector plasmid. The survivin expression was controlled by the hSyn1, neuron‐specific promoter. Enhanced green fluorescent protein (eGFP) was linked to the C‐terminus of survivin with the self‐cleaving T2A peptide. As a control, eGFP was cloned into an AAV ITR‐based vector plasmid.

### 2.2 Mice

All animal procedures were performed by the “Guide for the Care and Use of Laboratory Animals, 8th edition” of the National Institutes of Health (2011) and approved by the “Animal Care and Use Guidelines” of Kyung Hee University (approval number: KHSASP‐20‐232). We purchased 5XFAD mice (stock #034840) and B6SJLFL1 (stock #10012) mice for this study from Jackson Laboratory (Bar Harbor, ME, USA). Mice were bred and aged in our facility at 21°C–25°C and humidity at 50%–70% under a 12 h light/12 h dark cycle with free access to food and water. 7‐month‐old mice were used for the experiment.

### 2.3 Production and titration of AAV vectors

To produce AAV vectors, they were pseudotyped in AAV9 or AAVDJ capsids. HEK293T cells (ATCC, CRL‐3216) were transfected with pAAV‐ITR‐survivin, or pAAV‐ITR‐eGFP, pAAV2/9 encoding for AAV2rep and AAV9cap for AAV9 production and pAAVDJ encoding for AAV2rep and AAVDJcap for AAVDJ production, and helper plasmid. HEK293T cells were cultured in DMEM with 2% FBS. Recombinant pseudotyped AAV vector stocks were generated using PEI coprecipitation with PEIpro (Polyplus‐transfection) and triple‐transfection with plasmids at a molar ratio of 1:1:1 in HEK293T cells. After 72 h of incubation, cells were lysed and particles were purified by iodixanol (Sigma‐Aldrich, MO, USA) step‐gradient ultracentrifugation. The number of vector genomes was determined by quantitative PCR.

### 2.4 Isolation and culture of embryonic neural stem cells

Neural stem cells (NSCs) were obtained from the cortex and hippocampus of embryos on the embryonic day 14.5 fetuses as previously described.^25^ The cerebellum, midbrain, olfactory bulb, and blood vessels of the embryo were removed. The cortex and hippocampus tissues were immersed in cold Ca^2+^/Mg^2+^‐free Hanks' balanced salt solution (LB 203‐06; Welgene, Geongsan, Republic of Korea). Next, the tissues were incubated at 37°C for 20 min with DNase‐I (DN25; Sigma‐Aldrich, MO, USA) and Trypsin–EDTA solution (LS‐015‐08; Welgene, Geongsan, Republic of Korea). After stopping the reaction with fetal bovine serum (10082139; Gibco, MA, USA), cells were obtained by centrifugation. Cells were suspended as a single cell in an NSC medium: DMEM/F12 (LM‐002‐05; Welgene, Geongsan, Republic of Korea) supplemented with Penicillin/streptomycin (30010; Hyclone Laboratories, UT, USA), 1% N‐2 supplement (17502048; Gibco, MA, USA), 20 ng/mL EGF (PHG0311; Gibco, MA, USA), 20 ng/mL bFGF (PHG0368; Gibco, MA, USA), and 10% Insulin‐transferrin‐selenium‐sodium pyruvate (51,300,044; Gibco, MA, USA).^25^, ^26^ NSCs were seeded into 2 × 10^7^ cells on a 100 mm cell culture dish. After day 3, NSCs were sub‐cultured with a fresh medium for proliferation or differentiation.

### 2.5 Proliferation analysis

NSCs were cultured in an NSC medium: DMEM/F12 (LM‐002‐05; Welgene, Geongsan, Republic of Korea) supplemented with Penicillin/streptomycin (30010; Hyclone Laboratories, UT, USA), 1% N‐2 supplement (17502048; Gibco, MA, USA), 20 ng/mL EGF (PHG0311; Gibco, MA, USA), 20 ng/mL bFGF (PHG0368; Gibco, MA, USA), and 10% Insulin–transferrin–selenium–sodium pyruvate (51300044; Gibco, MA, USA) to proliferate into neurosphere as previously described.^25^ After the secondary sub‐culture, the single cells of NSCs were seeded into 1 × 10^4^ cells per well on a 96‐well plate. YM155 was treated 1 nM daily from the third day after subculture. Neurospheres were counted on the sixth day after seeding by using a K1‐Fluo confocal microscope (Nanoscope Systems, Daejeon, Republic of Korea). The diameters of neurospheres were measured using the ImageJ program.

### 2.6 Neural differentiation analysis

NSCs were cultured to differentiate into neuronal lineages as previously described, with some modifications.^27^, ^28^ The plate to be cultured was coated with 0.01% poly‐L‐lysine (P4707; Sigma‐Aldrich, MO, USA) overnight before subculture. After washing, NSCs, which were incubated for 3 days, were subcultured at 5 × 10^3^ cells/cm^2^ in a 24‐well plate for immunocytochemical staining and at 1 × 10^5^ cells/cm^2^ in a 6‐well plate for western blot assay. We changed medium for the neural differentiation medium: DMEM/F12 (LM‐002‐05; Welgene, Geongsan, Republic of Korea) supplemented with 50% neurobasal medium (21103049; Gibco, MA, USA), Penicillin/streptomycin (30010; Hyclone, UT, USA), 1% N‐2 supplement (17502048; Gibco, MA, USA), 20 ng/mL EGF (PHG0311; Gibco, MA, USA), 20 ng/mL bFGF (PHG0368; Gibco, MA, USA) and B27 supplement (17504044; Gibco, MA, USA). The medium was replaced once every 3 days for 14 days of incubation. YM155 was treated at 1 nM daily from the third day of subculture, and AAV‐GFP and AAV‐Survivin were treated once at a multiplicity of infection (MOI) of 10 in 1 × 10^5^ cells on the fourth day after subculture. An MOI of 1 was estimated with one infectious virus particle in 100 total viral particles determined by quantitative PCR.

### 2.7 Immunocytochemistry

To investigate neurogenesis from NSCs, cell immunochemical staining was performed. After removing the medium on the cover glass with the differentiated cells, it was washed twice with PBS, fixed with 4% paraformaldehyde at room temperature for 15 mi, and then washed three times with PBS‐Glycine. Incubating cells in 1% BSA (BSAS‐Au 0.1; BovoStar, Melbourne, *Australia*) solution in PBS at room temperature for 10 min, washed once, and reacted with doublecortin (DCX, ab18723; Abcam, Cambridge, UK) diluted at 1:500 overnight at 4°C. Alexa Fluor 594‐conjugated secondary antibody (A‐11012; Invitrogen, CA, USA) diluted at 1:1000 was incubated at room temperature in a light‐shielded for 1 h. After washing, the mounting solution was applied and then placed on a slide glass. Slide glasses were stored in a light‐shielded state. The slides were observed with a K1‐Fluo confocal microscope (Nanoscope Systems, Daejeon, Republic of Korea).

### 2.8 Stereotaxic injections

The viral vectors were injected by stereotaxic surgery as reported.^19^, ^29^, ^30^ The 7‐month‐old mice were randomly divided into the AAV‐GFP group and the AAV‐Survivin group. Mice were anesthetized by administration of a mixture of ketamine (100 mg/kg) and xylazine hydrochloride (10 mg/kg) in 0.9% NaCl.^31^ Brain coordinates were − 2.0 mm anterior–posterior, ±2.0 mm mediolateral, and − 1.75 mm dorsoventral to target DG. 2 μL of AAV9‐Survivin or AAV9‐GFP (1 × 10^11^ vg in 2 μL) were injected into the DG of the hippocampus. Virus microinjection was made through a 28‐gauge needle using a micro‐syringe pump controller at a constant rate of 0.2 μL/min.

### 2.9 BrdU injection and BrdU staining

5‐Bromo‐2′‐deoxynridine (BrdU, B5002; Sigma‐Aldrich, MO, USA) injection was performed as described previously.^25^ A month after stereotaxic injections, we treated BrdU intraperitoneally (50 mg/kg) for five consecutive days. BrdU was dissolved in 0.9% NaCl with 0.007 M NaOH. A month after the BrdU injection, the brain was cardiac‐perfused and fixed with 4% paraformaldehyde (PFA, P6148; Sigma‐Aldrich, MO, USA) in PB. The brain tissues were isolated and postfixed overnight at 4°C. After fixation, they were incubated in 30% sucrose in PBS at 4°C until equilibrated. Next, brains were serially cut into 25‐μm‐thick sections throughout the DG of the hippocampus region on a freezing microtome (Leica, Wetzlar, Germany) and stored at −20°C. In order to denature DNA, sections of brain tissue were incubated with 2 N HCL for 30 min at 37°C and neutralized with 0.1 M boric acid with three times washing. After neutralization, the sections were washed three times by PBS. Next, the sections were blocked in a blocking buffer with a PBS solution containing 3% normal goat serum (S‐1000; Vector Lab, CA, USA), 1% BSA (BSAS‐Au 0.1; BovoStar, Melbourne, *Australia*), and 0.4% Triton X‐100 (T9284‐500 M; Sigma‐Aldrich, MO, USA). After blocking, the sections were labeled by anti‐BrdU antibody (ab6326; Abcam, Cambridge, UK) diluted at 1:100. After 48 h' incubation, sections were visualized with secondary antibodies; Alexa Fluor 594‐conjugated secondary antibodies (A‐11007; Invitrogen, CA, USA). After the PBS washing, sections were applied to the fluorescence mounting medium (S3023; Dako, CA, USA) and covered by cover glasses. The brain tissues were observed with a K1‐Fluo confocal microscope (Nanoscope Systems, Daejeon, Republic of Korea).

### 2.10 Y‐maze

The spatial working memory function was tested using a symmetrical Y‐shaped maze as previously reported.^32^, ^33^ Each mouse was allowed to explore freely inside the maze for 8 min. The total number of arms entered and sequences were recorded for analysis. The spatial working memory function was evaluated by calculating the percentage of alternation that is the number of trials containing entries into all three arms divided by the maximum possible alternations (the total number of arms entered minus 2) × 100. The total number of entrances and exits that did not overlap with the three arms was not counted for analysis.

### 2.11 Morris Water Maze

The Morris Water Maze was performed, as previously reported.^34^ Using mice that had been trained for 1 day to escape to a platform below the surface of the water for the experiment. The platform was uniformly assigned to each mouse and set a fixed location throughout training in a room composed of cues on different sides. Three trials were conducted each day, and the mice were placed at a different starting point for each trial. The training was conducted for 7 days, the escape time to the platform was recorded, and the average value of three trials per day was used as data for each day. On the 8th day of training, a probe test was performed for 60 s without the platform. The probe test was conducted for measuring the total distance for a swim, the number of platform zone crosses, and the time that the mouse spent in the quadrant with the platform zone.

### 2.12 Immunofluorescence

To observe the effects of survivin overexpression in DG, immunohistochemical staining was conducted for DCX, 6E10, and GFAP respectively. After the behavioral test, all 9‐month‐old mice were anesthetized. 5XFAD and wild‐type (WT) female mice were immediately cardiac‐perfused with 4% PFA (in PB). Next, brain tissues were isolated and post‐fixed overnight at 4°C After fixation, they were incubated in 30% sucrose in PBS at 4°C until equilibrated. Next, brains were serially cut into 25 μm‐thick sections throughout the DG of the hippocampus region on a freezing microtome (Leica, Wetzlar, Germany) and stored at −20°C. The brain sections were sequentially incubated with a PBS solution containing 3% normal goat serum (S‐1000; Vector Lab, CA, USA), 1% BSA (BSAS‐Au 0.1; BovoStar, Melbourne, *Australia*), and 0.4% Triton X‐100 (T9284‐500 M; Sigma‐Aldrich, MO, USA) and labeled anti‐NeuN (MAB377; Milipore Corp., MA, USA) diluted at 1:300, anti‐DCX (ab18723; Abcam, Cambridge, UK) diluted at 1:500, anti‐6E10 (803,001; BioLegend, CA, USA) diluted at 1:500 or anti‐GFAP (ab4674; Abcam, Cambridge, UK) diluted at 1:1000 and counterstained with DAPI. Sections were visualized with secondary antibodies; Alexa Fluor 594‐conjugated secondary antibodies (A‐11012; Invitrogen, CA, USA) and Alexa Fluor 488‐conjugated secondary antibodies (A11001; Invitrogen, CA, USA). After the PBS washing, sections were applied to the fluorescence mounting medium (S3023; Dako, CA, USA) and covered by cover glasses. ImageJ was used to estimate the number of DCX^+^, NeuN^+^, and % area of 6E10^+^ and GFAP^+^ cells in the dentate gyrus of the hippocampus. The brain tissues were observed with a K1‐Fluo confocal microscope (Nanoscope Systems, Daejeon, Republic of Korea).

### 2.13 Western blot

Western blot was performed in previous studies.^35^ RIPA buffer (89,901; Thermo Fisher Scientific, MA, USA) and protease/phosphate inhibitors (78,444; Thermo Fisher Scientific, MA, USA) were used for the cell lysis step. Equal amounts of protein samples (30 or 50 μg) were prepared and boiled with protein 5× sample buffer (EBA‐1052; Elpis biotech, Taejon, Korea) at 95°C for 5 min. Protein samples were separated on sodium dodecyl sulfate‐polyacrylamide gel and transferred to Polyvinylidene difluoride membranes by electrophoresis. The membranes were washed using 0.1% Tween 20 in TBS buffer (TBST) and then blocked with TBST solution containing 5% BSA (BSAS‐Au 0.1; BovoStar, Melbourne, *Australia*). Thereafter, the membranes were incubated with DCX antibody (ab18723; Abcam, Cambridge, UK) or NeuN antibody (MAB377; Millipore, MA, USA) or *β*‐actin antibody (sc‐4778HRP; Santa Cruz Biotechnology, TX, USA) overnight at 4°C. All primary antibodies were used at 1:1000 dilution except β‐actin at 1:5000. After washing with TBST four times for 5 min, horse radish‐peroxidase‐conjugated secondary antibodies at 1:5000 were reacted for 1 h. The protein expressions were detected by using an ECL reagent (BR170‐5061; BIO‐RAD Laboratories, CA, USA) and captured by Solo6S EDGE (Vilber Lourmat Sté, Collégien, France). The levels of protein expression were comparatively analyzed using the Image‐J program.

### 2.14 Quantitative RT‐PCR

RNA extraction and qRT‐PCR were performed in previous studies.^36^ RNA extraction was conducted to use for Kit Hybrid‐R™ (305–101; GeneAll, Seoul, Republic of Korea) and the concentration of each RNA sample was measured using a Nanodrop ND‐1000 spectrophotometer (Thermo Fisher Scientific). cDNA was synthesized using TOPscript™ RT DryMIX (RT200; Enzynomics, Daejeon, Republic of Korea) at a final concentration of 3 or 5 μg. 3% forward and reverse primer (10 pM), 4% SYBR Green PCR master mix, and 4.65% RNase‐free water was used for synthesis. Primers were made by Cosmo Genetech (Seoul, Republic of Korea). GAPDH was used as an internal control. The following primers were used: Prox1 (Gene ID: 19130; forward 5′‐GACATGAACAATCCCTTTGC‐3′, reverse 5′‐GCTTCCTTGCGTGTAAGTGT‐3′) Neurogenin‐2 (Gene ID: 11924; forward 5′‐CTC GCCAGGGACTGTATCTA‐3′, reverse 5′‐CCACCTCTGCTCTGTGAAGT‐3′), NeuroD1 (Gene ID: 18012, forward 5′‐ CGATTAGAGGCACGTCAGTT‐3′, reverse 5′‐TTCTTCCAAAGGCAGTAACG‐3′), GAPDH (Gene ID: 14433; forward 5′‐TGAATACGGCTACAGCAACA‐3′, reverse 5′‐AGGCCCCTCCTGTTATTATG‐3′).

### 2.15 Cell quantification

For the quantification of cells expressing specific markers in immunocytochemistry, we repeated the analysis using at least three different mouse NSC samples and calculated the average cell count from three fields of slide. The number of positive cells was determined using ImageJ. For the quantification of cells with markers in immunohistochemistry, we randomly selected three to four brain sections per mice (spaced apart from Bregma −1.3 to −2.7). The number of positive cells was quantified using ImageJ.

### 2.16 Statistical analysis

All data are presented as mean ± standard error of the mean and all statistical analyses were performed in GraphPad Prism (Version 8.0a; GraphPad Software Inc). The differences between the groups were analyzed with the Student's *t*‐test. One‐way analysis of variance (ANOVA) via Tukey's post hoc test or one‐way ANOVA analysis of variance via Tukey's multiple comparisons test or two‐way analysis of variance and Tukey's post hoc test was used for multiple group comparisons. All cell experiments were repeated three to four times, and in the case of animal experiments, *n* represents the number of mice. A value of *p < 0.05 indicates a statistically significant difference. In all cases, the standard deviation is reported and significance values are denoted as follows; *p < 0.05, **p < 0.01, ***p < 0.001, **** *p* < 0.0001.

## 3 RESULTS

### 3.1 The effect of survivin on neurogenesis in NSCs

**FIGURE 1:**
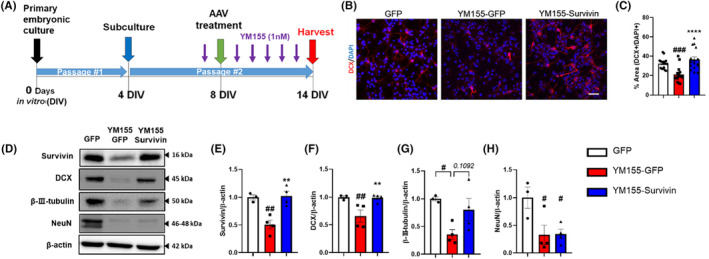
The expression level of survivin in NSCs regulates neurogenesis. (A) Timeline of primary mouse embryonic cultures and in vitro experiment for neural differentiation analysis. (B, C) Immunocytochemical analysis of neurogenesis from NSCs in vitro. (B) Representative images of DCX (Alexa Fluor 594) and DAPI (blue) from NSCs in vitro (scale bar = 30 μm). (C) Graphs showing the comparison of percentages of DCX^+^DAPI^+^ cells per DAPI^+^ cells (*n* = 3). (D) Representative immunoblot images of differentiated NSCs in vitro. (E–H) Quantification of protein levels was analyzed using ImageJ and the normalized β‐Actin level (*n* = 3–4). Data were analyzed using one‐way analysis of variance and Tukey's post hoc test (error bars: Sem). **p < 0.01, ****p < 0.0001 versus YM155‐GFP and ^#^ *p* < 0.05, ^##^ *p* < 0.01, ^###^ *p* < 0.001 versus GFP control.

We investigated the impact of survivin on proliferation in NSCs. We initially evaluated the effects of reducing survivin levels using YM155, a well‐known survivin inhibitor that inhibits survivin promoter activity, on NSCs. To assess the impact of this inhibition, we incubated neurospheres for proliferation analysis and compared the quantity and size of the neurospheres in both the control group and the group treated with YM155. Our findings revealed that decreased expression of survivin following YM155 treatment inhibited the proliferation of NSCs (Figure S1). Next, we assessed the changes in survivin expression levels and neuronal lineage in embryonic NSCs treated with YM155 and AAVDJ‐survivin (Figure 1A). The YM155‐GFP group received AAVDJ‐GFP after YM155 exposure and the YM155‐survivin group received AAVDJ‐Survivin after YM155 exposure. After 14 d of neuronal lineage differentiation from embryonic NSCs, we analyzed the expression of doublecortin (DCX), an immature neuronal marker, in differentiated neural progenitor cell (NPCs) progeny. Our findings demonstrated that the number of DCX^+^DAPI^+^ cells decreased in the YM155‐GFP group, whereas the YM155‐Survivin group showed a recovery in the number of DCX^+^DAPI^+^ cells (Figure 1B,C). In addition, we observed a significant decrease in DCX protein levels upon the suppression of survivin expression (Figure 1D). However, when the reduced survivin expression was restored by AAV‐Survivin treatment, there was a marked recovery in neurogenesis (Figure 1D,F). There was a trend for an increase of β‐III‐tubulin (immature neuron and mature neuron marker) expression in the YM155‐Survivin group compared to YM155‐GFP, but no statistically significant change in NeuN (mature neuron marker) was observed between YM155‐GFP and YM155‐Survivin (Figure 1D,G,H). Notably, the generation of mature neurons may require more time in an in vitro model, which may be influenced by the specific combination of media used.^37^, ^38^ Collectively, these data suggest that survivin regulates neurogenesis.

### 3.2 Survivin overexpression in 5XFAD increases neurogenesis

**FIGURE 2:**
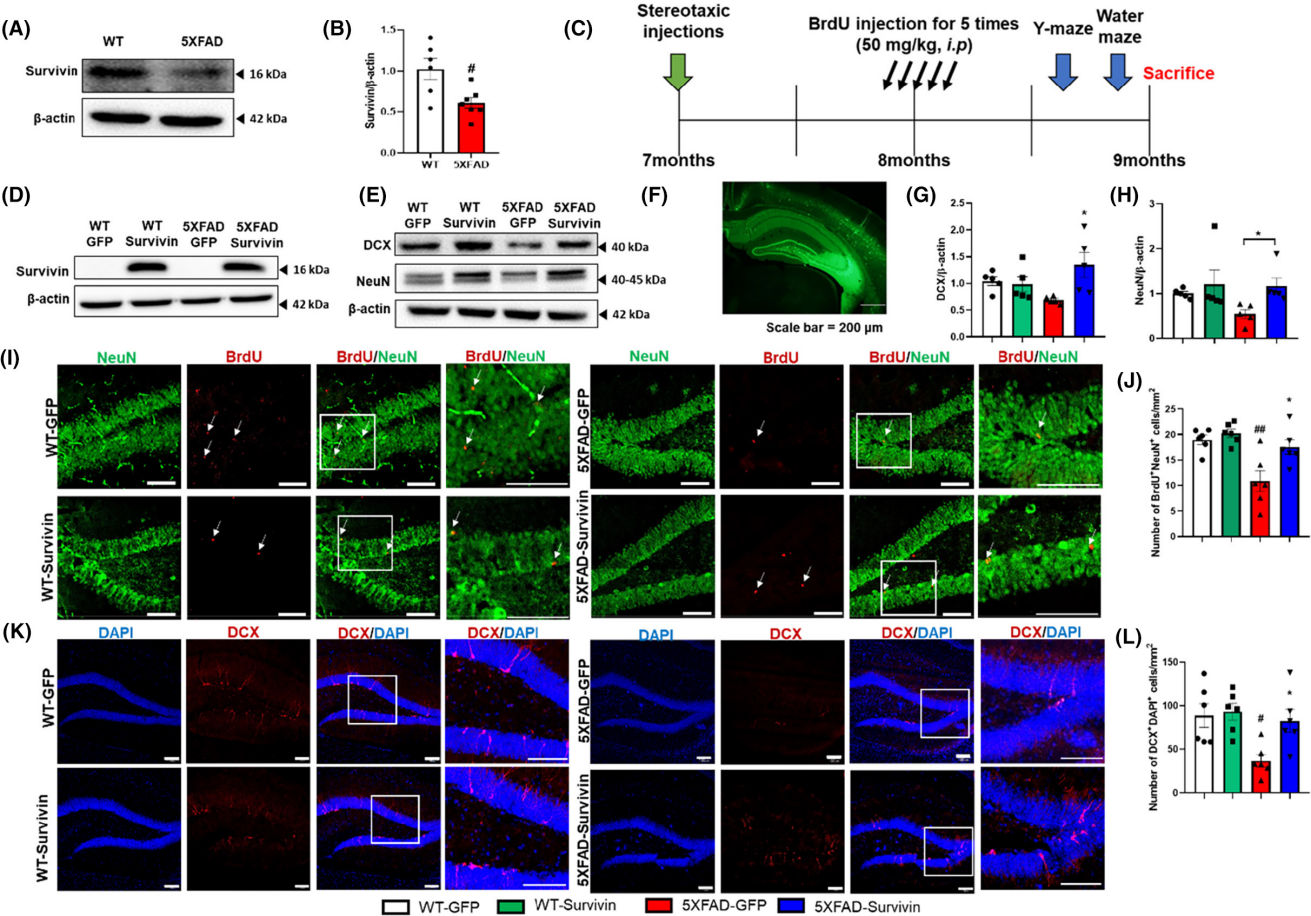
Survivin overexpression in 5XFAD mice enhances neurogenesis of immature and mature neurons. (A, B) Representative images (A) and quantifications (B) of immunoblot analysis of hippocampus for survivin expression in 9‐month‐old WT and 5XFAD mice (*n* = 4 per group). Data were analyzed using Student's *t*‐test. ^#^ *p* < 0.05 versus WT. (C) Timeline of the in vivo experiment with AAV injection. (D) The AAV2/9‐Survivin or AAV2/9‐GFP was injected in the DG of the hippocampus through stereotaxic surgery, and survivin was overexpressed in the AAV2/9‐Survivin injection groups. (E) GFP was observed in the mouse hippocampus section (scale bar = 200 μm). (F–H) Western blot analysis of hippocampus for DCX and NeuN expression. (G) DCX expression in the hippocampus was increased in 5XFAD‐Survivin compared with 5XFAD‐GFP. Data were analyzed using one‐way analysis of variance and Tukey's post hoc test (*n* = 5–6 per group; error bars: SEM). *p < 0.05 versus 5XFAD‐GFP. (H) NeuN expression in the hippocampus was increased in 5XFAD‐Survivin compared with 5XFAD‐GFP. Data were analyzed using Student's *t*‐test. *p < 0.05 versus 5XFAD‐GFP (*n* = 5–6 per group; error bars: SEM). (I) Representative images of mouse DG slices stained with BrdU (Alexa Fluor 594) antibody and NeuN (Alexa Fluor 488) antibody (scale bar = 100 μm). (J) Quantification of BrdU^+^ and NeuN^+^ double‐stained cells per mm^2^ DG region was performed by counting in three sections per mouse throughout the hippocampus. (K) Representative images of mouse DG slices stained with DCX (Alexa Fluor 594) antibody and DAPI (blue) (scale bar = 100 μm). (L) DCX^+^DAPI^+^ cells were counted in three sections per mouse throughout the hippocampus. Data were analyzed using a one‐way analysis of variance and Tukey's post hoc test (*n* = 6 per group; error bars: SEM). *p < 0.05 versus 5XFAD‐GFP. ^#^ *p* < 0.05, ^##^ *p* < 0.01 versus WT‐GFP.

To explore the potential role of survivin expression in regulating neurogenesis in AD, we conducted experiments using 5XFAD mice, a well‐established model that exhibits deficits in neurogenesis and cognitive performance.^39^, ^40^ To investigate the connection between neurogenesis and survivin expression in AD, we performed immunohistochemical and immunoblot analyses of neurogenesis in the hippocampi of 9‐month‐old WT and 5XFAD mice. Consistent with previous studies, we observed a reduction in the number of immature neurons in the DG of 5XFAD mice compared to that in WT mice (Figure S2). Furthermore, we found that survivin expression was decreased in the hippocampus of 5XFAD mice, suggesting a potential role for survivin in influencing hippocampal neurogenesis (Figure 2A,B).

To determine whether survivin affects neurogenesis in AD, the AAV expressing survivin (AAV2/9‐Survivin) were stereotaxically injected into the DG of 7‐month‐old WT and 5XFAD mice (Figure 2C). Two months after surgery, we assessed the expression level of survivin using immunoblot analysis and verified the stereotaxic injection site by examining GFP expression. As shown in Figure 2D,E, AAV2/9‐Survivin treatment resulted in a significant increase in the protein expression of survivin. In addition, the numbers of immature and mature neurons were higher in the 5XFAD‐survivin group compared to in the 5XFAD‐GFP group (Figure 2F,G,H). The number of BrdU^+^ and NeuN^+^ double‐stained cells in the hippocampus increased in the 5XFAD‐Survivin group (Figure 2I,J). The number of DCX^+^ and DAPI^+^ double‐stained cells in the 5XFAD‐survivin group was significantly higher than in the 5XFAD‐GFP group (Figure 2K,L). We also performed immunostaining for GFAP; however, there was no significant difference between the 5XFAD‐GFP and 5XFAD‐Survivin groups, indicating that survivin overexpression did not have a substantial impact on inflammatory cells (Figure S3). Next, we evaluated whether survivin overexpression affects Aβ deposition. In our study, survivin overexpression did not significantly influence Aβ accumulation in the 5XFAD mouse brain (Figure S4). Taken together, these results suggest that survivin promotes neurogenesis without significantly altering inflammation or Aβ deposition in 5XFAD mice.

### 3.3 Proneural genes in the hippocampus increase in Survivin overexpressed 5XFAD mice

**FIGURE 3:**
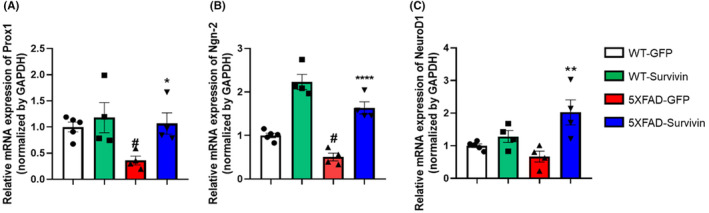
Expression of proneural genes was upregulated in survivin‐overexpressed 5XFAD mice. (A–C) The mRNA expression of proneural genes was assessed using qRT‐PCR in the hippocampus. Data were analyzed using one‐way analysis of Variance and Dunnett's multiple comparisons tests (*n* = 4–5 per group; error bars: SEM). *p < 0.05, **p < 0.01, ****p < 0.0001 versus 5XFAD‐GFP and ^#^ *p* < 0.05 versus WT‐GFP.

Previous studies have shown that survivin regulates the expression of prospero‐related homeodomain transcription factor 1 (Prox1). Prox1 is a transcription factor that plays a significant role in the regulation of NSCs differentiation, specifically in guiding NSCs toward neuronal differentiation during neurogenesis.^20^, ^21^, ^22^ Prox1 is expressed in neuroblasts, immature neurons, and mature neurons.^21^, ^22^, ^23^ Based on these results, we examined the Prox1 mRNA expression. Notably, the mRNA expression of Prox1 in the 5XFAD‐GFP group was approximately 2‐fold lower than that in the WT‐GFP group (Figure 3A). However, in the 5XFAD‐survivin group, the expression level of Prox1 was 2‐fold higher compared than in the 5XFAD‐GFP group (Figure 3A). Previous reports have indicated that Prox1 promotes Wnt/β‐catenin signaling, which, in turn, regulates the expression of proneural genes such as Neurogenin‐2 (Ngn‐2) and NeuroD1.^41^, ^42^, ^43^ In our study, we observed that both Ngn‐2 and NeuroD1 mRNA levels were higher in the hippocampus of 5XFAD‐Survivin mice compared than in 5XFAD‐GFP mice. The mRNA expression level of Ngn‐2 in the 5XFAD‐Survivin group was 2‐ to 2.5‐fold higher than that in the 5XFAD‐GFP group (Figure 3B). Additionally, the mRNA expression level of NeuroD1 in the 5XFAD‐Survivin group was 2‐fold higher than that in the 5XFAD‐GFP group (Figure 3C). These results collectively suggest that survivin regulates the expression of Prox1, which in turn enhances the expression of proneural genes, such as Ngn‐2 and NeuroD1. This regulatory mechanism contributes to the restoration of neurogenesis in the DG of 5XFAD mice.

### 3.4 Survivin ameliorates learning and memory deficits in 5XFAD mouse model

**FIGURE 4:**
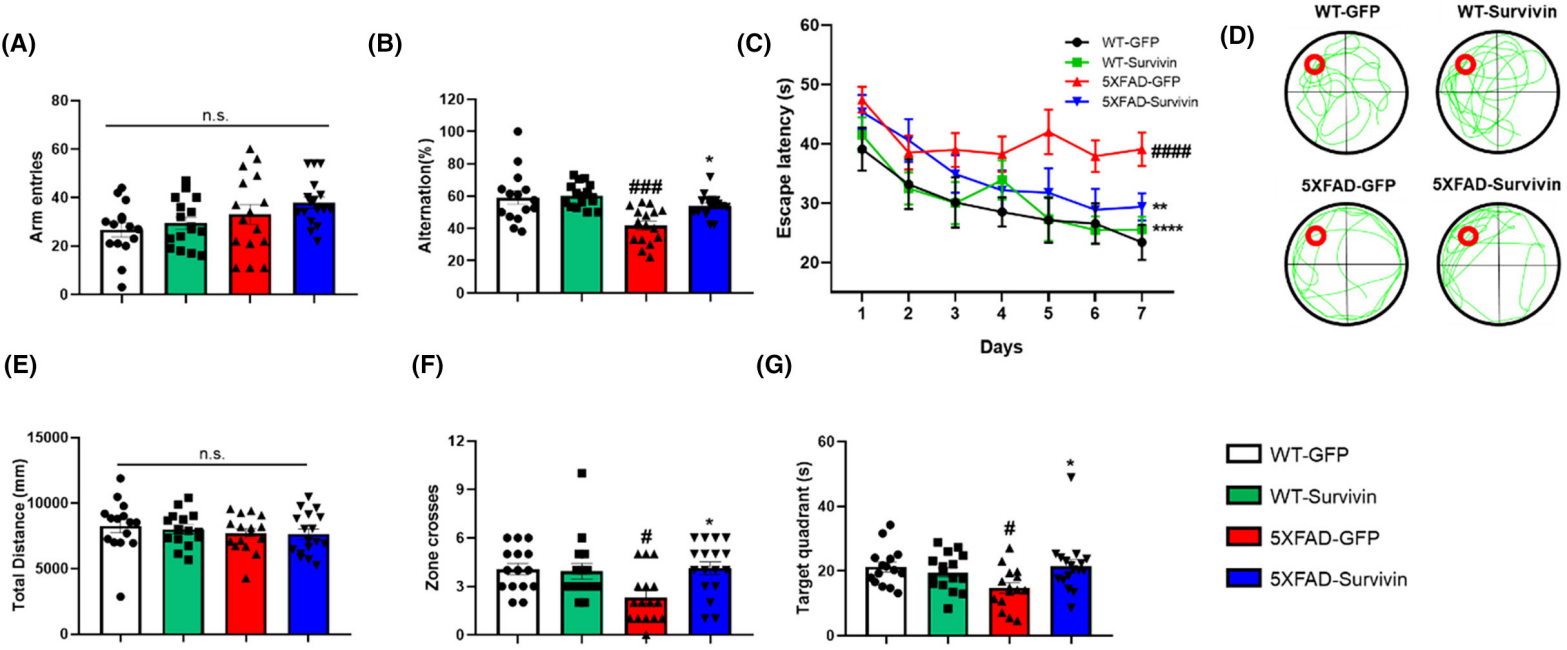
Survivin overexpression promotes cognitive function in 5XFAD mice. (A, B) Working memory function was assessed using the Y maze (*n* = 15–17 per group; error bars: SEM). (A) The number of moves in the Y‐shaped maze is schematized. (B) In the total number of movements, the ratio of the number of times of entry into the three arms without overlapping in the Y‐shaped maze was compared. (C) Spatial learning and memory were analyzed using Morris water maze estimating the time to reach a hidden platform for 7 consecutive days. Data were analyzed using two‐way analysis of variance and Tukey's post hoc test. **p < 0.01, ****p < 0.0001 versus 5XFAD‐GFP group and ^####^ *p* < 0.0001 versus WT‐GFP group. (D‐F) On day 8, the representative swimming paths of groups the platform was removed (D), the total moving distance (E), the number of times the platform point was crossed (F), and the time of stay in the quadrant where the platform was located (G) were measured for 1 minute (*n* = 15–17 per group; error bars: SEM). Data were analyzed using one‐way analysis of variance and Tukey's post hoc test. *p < 0.05 versus 5XFAD‐GFP group and ^#^ *p* < 0.05 versus WT‐GFP group.

To investigate the potential effects of survivin overexpression on cognitive function, we conducted behavioral tests using the Y‐maze and Morris water maze (MWM) (Figure 4). In the Y‐maze test, no significant differences were observed in the number of moves between the groups. However, the ratio of entries into the three arms without overlap significantly increased in the 5XFAD‐Survivin group compared to the 5XFAD‐GFP group, indicating an improvement in short‐term memory and cognitive function (Figure 4A,B). Additionally, the MWM test was conducted to determine whether spatial memory and cognitive function were improved by AAV‐survivin. On the first day, the mice in all groups reached the hidden platform within a similar amount of time. However, on the seventh day, the 5XFAD‐survivin group exhibited a significantly shorter escape time to reach the hidden platform than did the 5XFAD‐GFP group, indicating improved memory and spatial learning abilities (Figure 4C). During the probe test on day 8, when the platform was removed, there was no significant difference in the distance moved (Figure 4E). However, the 5XFAD‐survivin group demonstrated a higher number of crossings in the zone where the platform was previously located (Figure 4D,F). In addition, the 5XFAD‐survivin group spent more time in the quadrant of the pool that belonged to the platform than did the 5XFAD‐GFP group (Figure 4G). Taken together, these data indicated that survivin overexpression rescued cognitive function and spatial memory in 5XFAD mice.

## 4 DISCUSSION

Although there is currently no treatment for stopping or reversing AD, promoting hippocampal neurogenesis has emerged as a crucial strategy for improving cognitive function. Accumulating evidence suggests a strong association between the promotion of neurogenesis and AD treatment.^8^, ^44^, ^45^ In the present study, we demonstrated that survivin enhances neurogenesis and cognitive function in AD. NSCs were incubated with AAV‐GFP or AAV‐Survivin after YM155, a survivin inhibitor, exposure. YM155 with AAV‐GFP treatment suppressed the generation of immature neurons, whereas YM‐155 with AAV‐survivin treatment induced the generation of immature neurons by restoring survivin expression. Additionally, our results indicated a decrease in survivin expression in AD and its association with impaired neurogenesis. We also showed that increased survivin expression enhances hippocampal neurogenesis in vivo by increasing the expression of proneural genes. Moreover, the overexpression of survivin in 5XFAD mice led to improved cognitive performance.

Prior investigations have explored various strategies to restore neurogenesis in AD, including the enhancement of Wnt/β‐catenin signaling and the overexpression of transcription factors involved in neurogenesis regulation.^46^, ^47^ Consistent with previous studies, our data revealed that AAV‐Survivin promotes neurogenesis by upregulating the expression of transcription factors, such as Prox1, NeuroD1, and Ngn‐2. Prox1, which is regulated by survivin, and plays a crucial role in controlling embryonic and adult neurogenesis by inducing neuronal lineage and suppressing astrocyte expression.^48^ Previous studies have demonstrated that inducing Prox1 expression enhances neurogenesis, improves hippocampal function, and enhances contextual memory.^23^, ^49^ NeuroD1 is involved in the development of newborn neurons by dividing NSCs in normal mice.^11^ However, when NeuroD1 alone was overexpressed, AD mice did not generate newborn neurons.^11^ In our study, we observed that survivin overexpression in 5XFAD mice led to an increase in the expression of NeuroD1 and a significant increase in the number of immature neurons. These results differ from those of a previous study, possibly because of the involvement of other transcription factors. Therefore, survivin overexpression may coordinate neurogenesis in AD. Ngn‐2 regulates neurogenesis by facilitating differentiation of NSCs into neurons. Previous studies have indicated that Ngn‐2 activates NeuroD1 and directly promotes neuroblast expression, which affects neuronal development.^46^, ^50^ Consistent with these findings, we observed that survivin overexpression in 5XFAD mice significantly enhanced the expression of Ngn‐2 and NeuroD1. Therefore, it is reasonable to hypothesize that survivin overexpression in AD enhances neurogenesis by upregulating the expression of transcription factors, such as Prox1, Ngn‐2, and NeuroD1.

In conclusion, our study provides evidence that decreased survivin expression contributes to the decline in neurogenesis in AD, while survivin overexpression in the hippocampus of AD mouse models promotes neurogenesis by regulating the expression of transcription factors. Considering that reduced neurogenesis is observed as a symptom in patients with AD, investigating neurogenesis represents a promising avenue for AD treatment. Consequently, targeting survivin as a promoter of neurogenesis may provide fundamental insights into the development of potential therapeutic strategies.

## CONFLICT OF INTEREST STATEMENT

The authors declare no conflict of interest.

## Supporting information


Figures S1–S4

Appendix S1

## Data Availability

The data that support the findings of this study are available from the corresponding author upon reasonable request.
