# Supplementary material for: Survivin enhances hippocampal neurogenesis and cognitive function in Alzheimer's disease mouse model
Source: CNS Neurosci Ther. 2023 Oct 30;30(4):e14509. doi: 10.1111/cns.14509 (PMC11017468; doi:10.1111/cns.14509)
Supplement: Supplementary file 1 — Figures S1–S4 [file CNS-30-e14509-s001.docx]

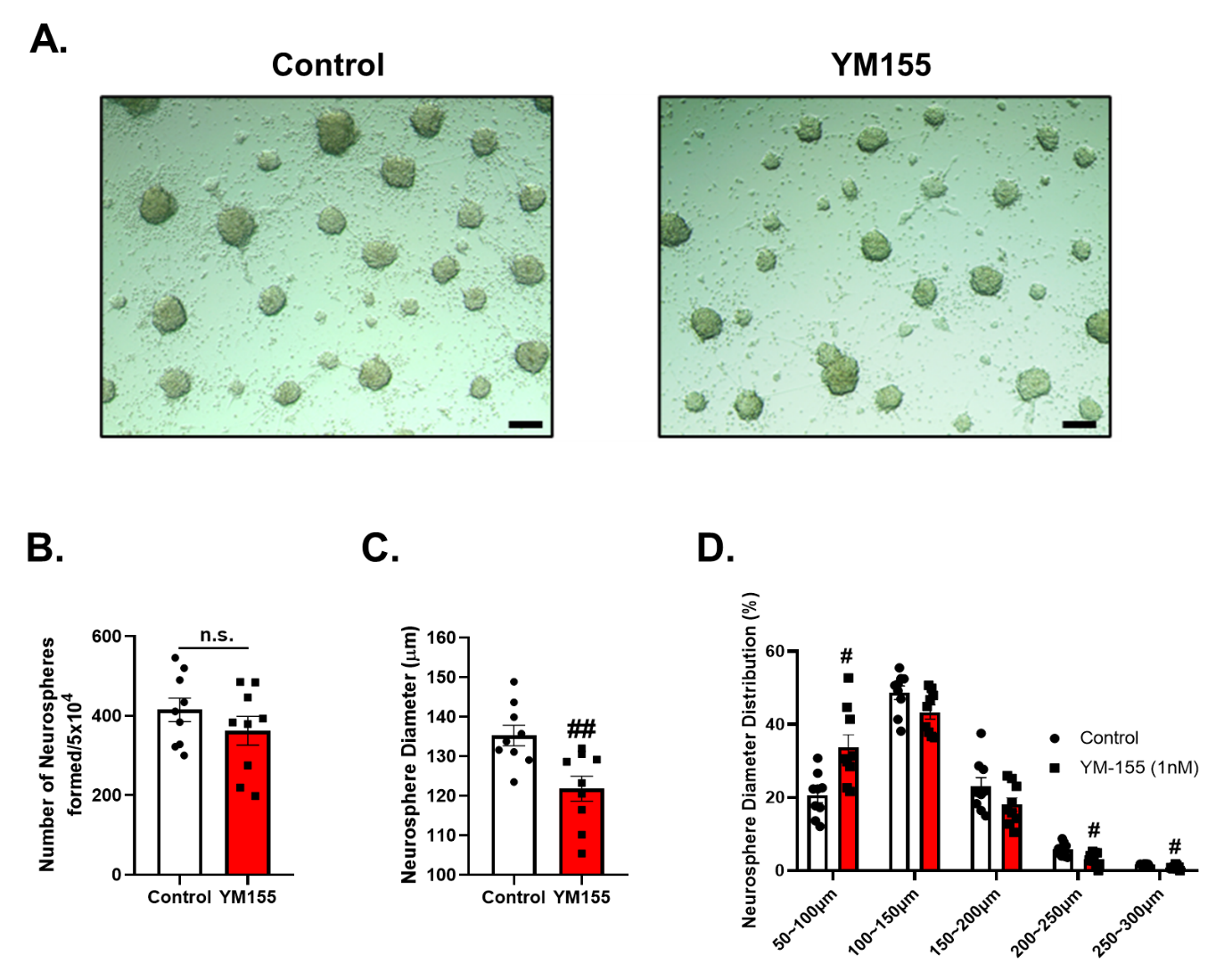


**Supplementary figure 1. Reduced survivin expression inhibits proliferation of NSCs**. (**A**) Representative images of neurospheres cultured from NSCs *in vitro* (scale bar = 100 μm). (**B-D**) Graphs showing the comparison of the number (**B**), diameter (**C**) and distribution of diameter of neurosphere formed (**D**). Data were analyzed by Student’s t-test or Two-way ANOVA analysis of variance with Bonferroni test (n = 3; error bars: SEM). ^#^*p* < 0.05, ^##^*p* <0.01 *versus* Control.

**
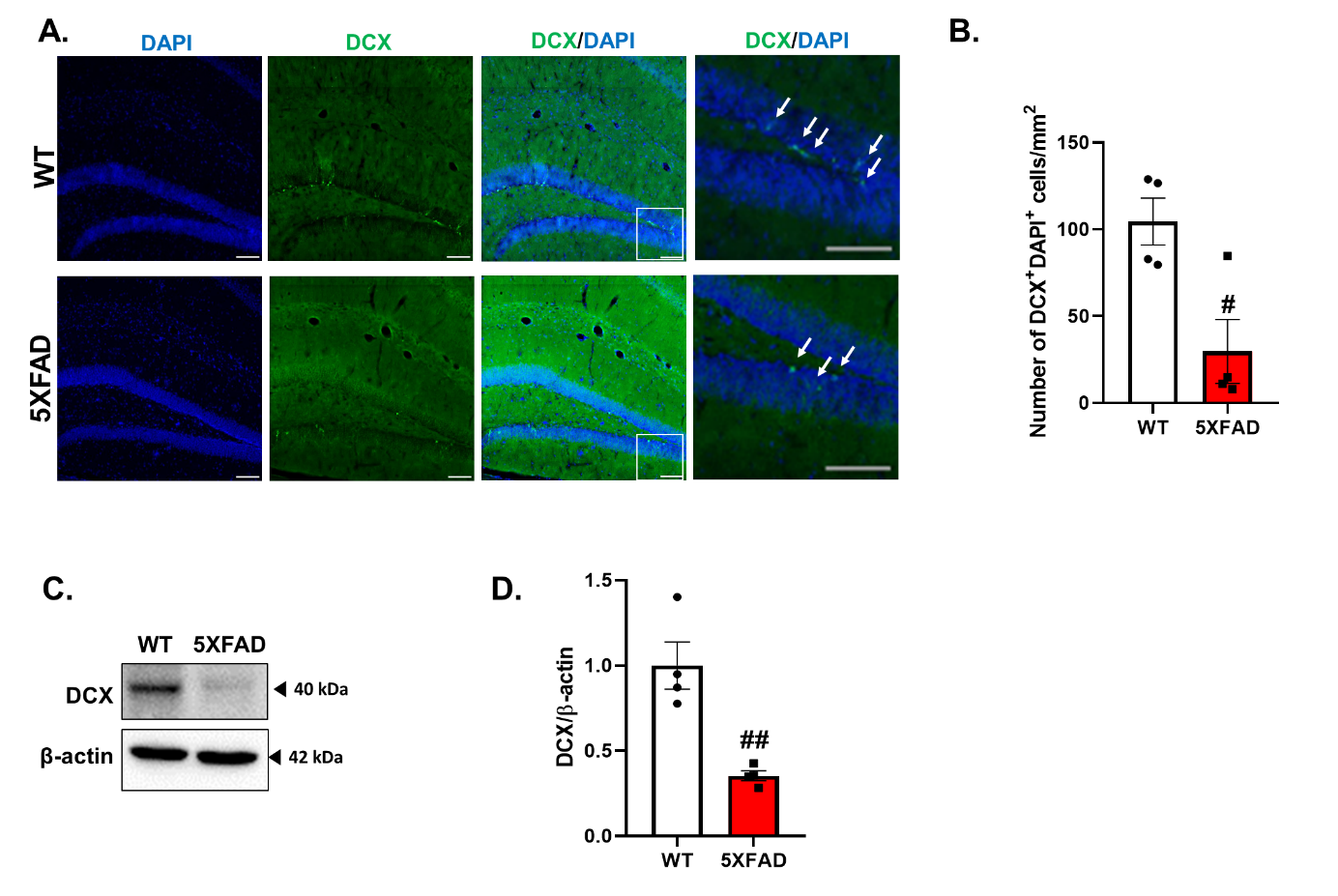
**

**Supplementary Figure 2. Neurogenesis is reduced in the hippocampus of 9-month-old 5XFAD mice.** (**A**) Representative images of DCX^+^ cells in the hippocampus of 9-month-old WT and 5XFAD mice (scale bar = 100 μm). (**B**) Graphs showing the comparison of percentage of DCX^+^DAPI^+^ cells (n = 3). (**C-D**) Representative immunoblot images (**C**) and quantifications (**D**) for DCX in the hippocampus (n = 4). Data were analyzed by student’s t-test (error bars: SEM). ^#^*p* < 0.05, ^##^*p* <0.01 *versus* WT.

**
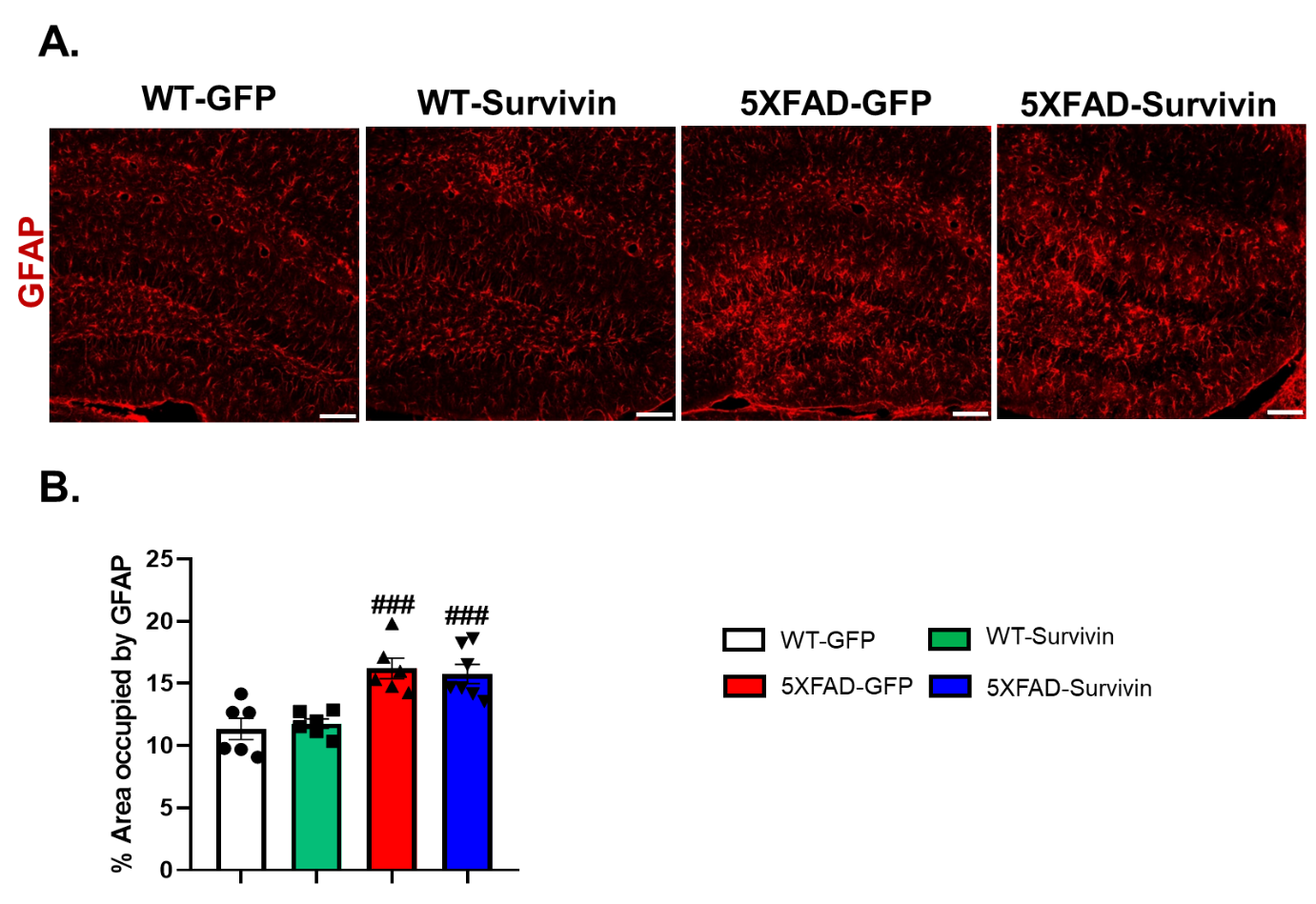
**

**Supplementary figure 3. Survivin over-expression did not affect astrocyte activation in 5XFAD mice.** (**A**) Representative immunofluorescence images of GFAP (scale bar = 100 μm). (**B**) Graphs showing the comparison of percentage of GFAP^+^ cell areas (n = 6-7 per group). Data were analyzed using one-way analysis of Variance and Tukey’s post hoc test (error bars: SEM). ^###^*p* < 0.001 *versus* WT-GFP.

**
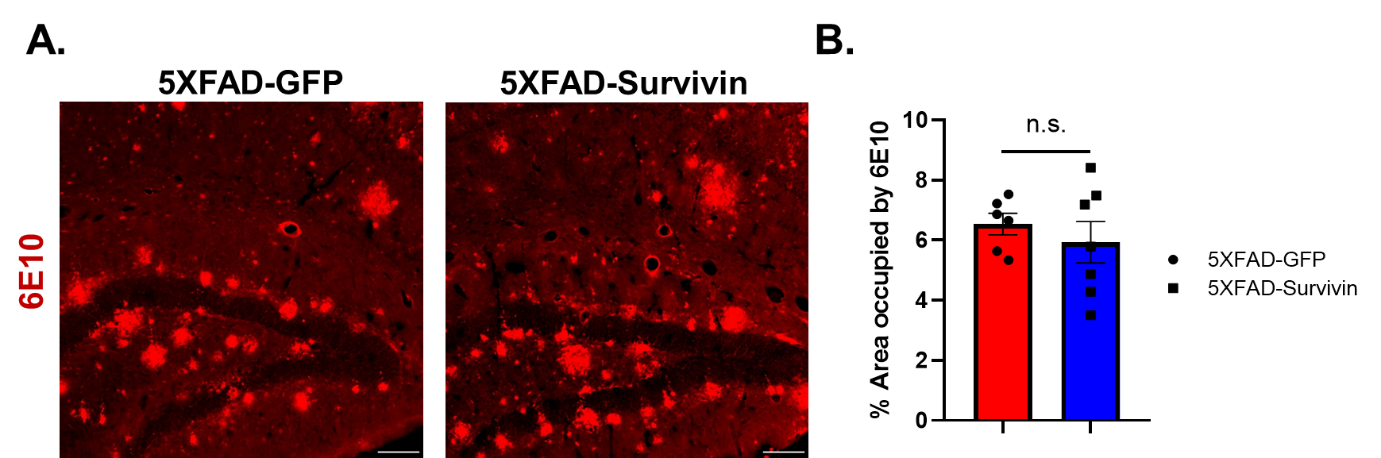
**

**Supplementary Figure 4. Survivin overexpression did not affect Aβ deposition in 5XFAD mice.** (**A**) Representative immunofluorescence images of 5XFAD-GFP and 5XFAD-Survivin mouse brain slices stained with 6E10 antibody (scale bar = 100 μm). (**B**) Graphs showing the comparison of percentage of 6E10^+^ areas (n = 6-7 per group). Data were analyzed by Student’s t-test (error bars: SEM).
