# Supplementary figures and images for: Survivin enhances hippocampal neurogenesis and cognitive function in Alzheimer's disease mouse model
Source: CNS Neurosci Ther. 2023 Oct 30;30(4):e14509. doi: 10.1111/cns.14509 (PMC11017468; doi:10.1111/cns.14509)

Figure 1D.

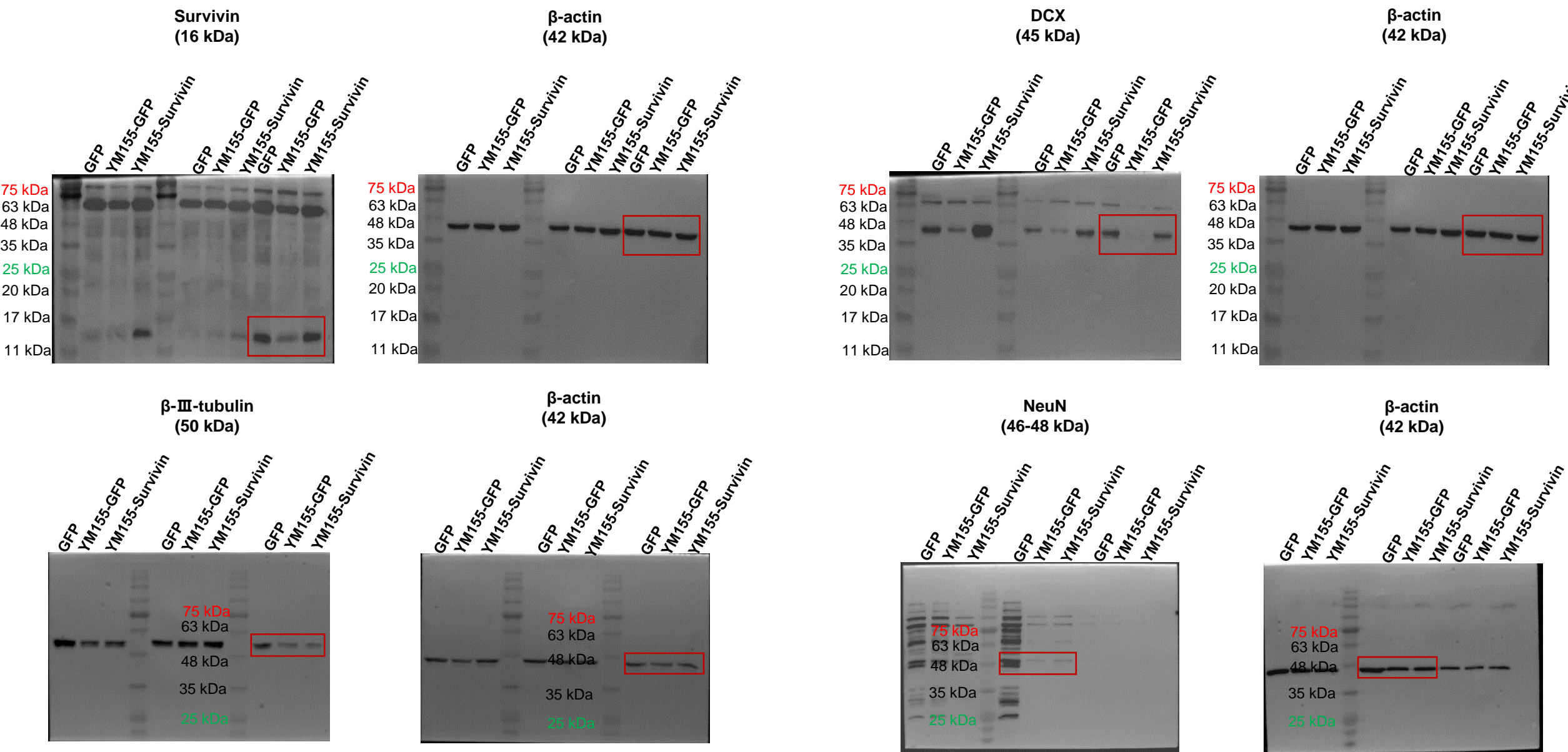

Figure 2A.

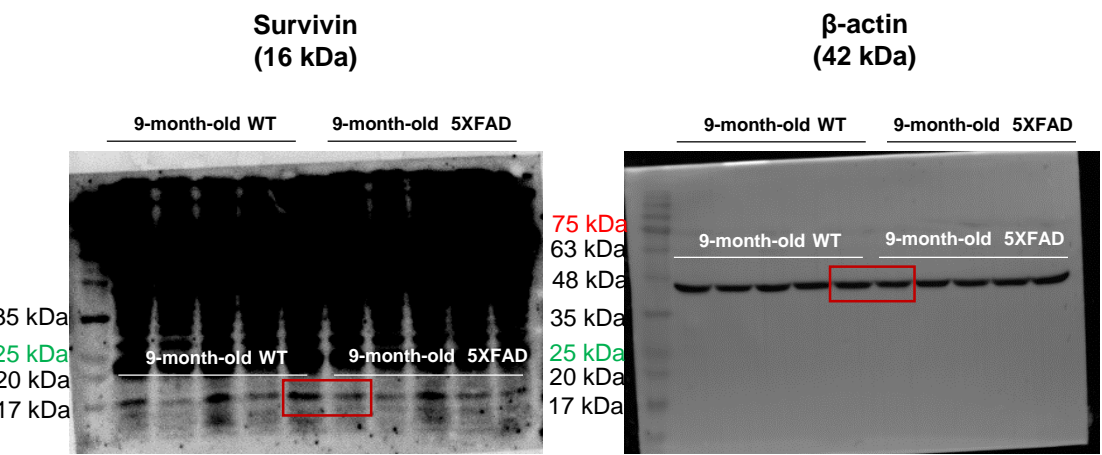

Figure 2D.

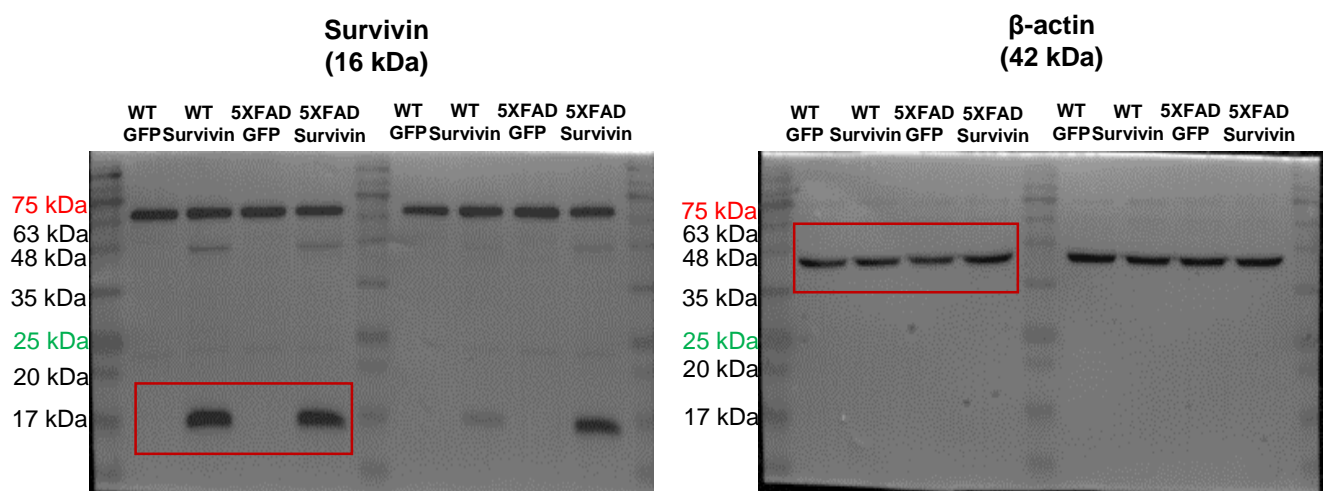

Figure 2E.

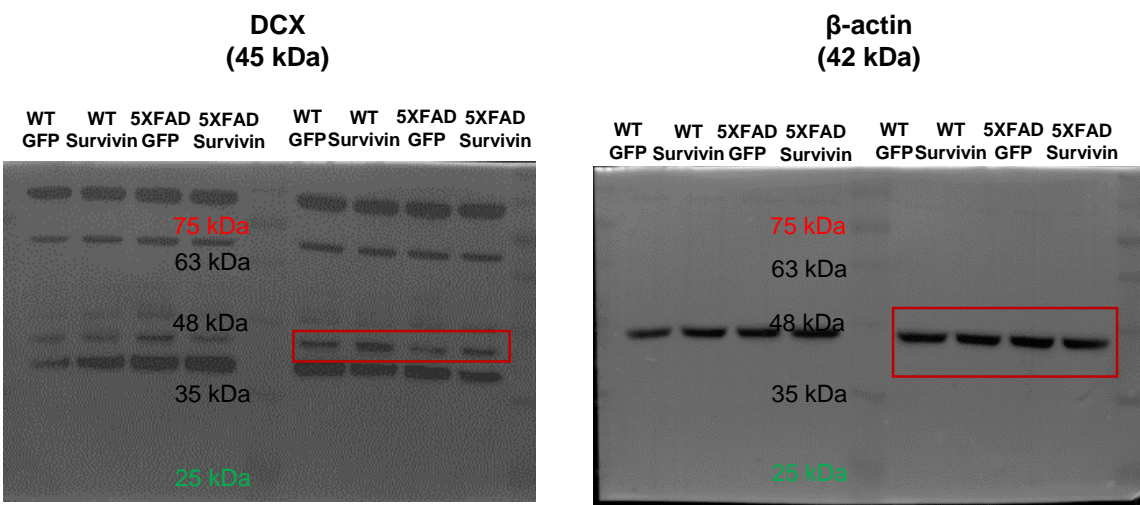

Figure 2E.

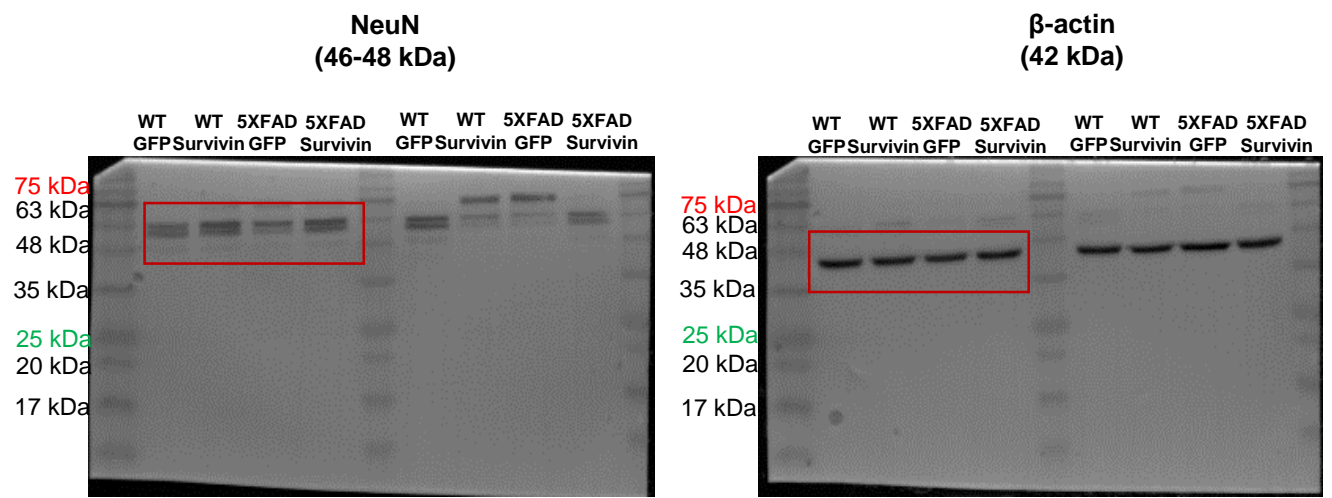

Supplementary figure 2C.

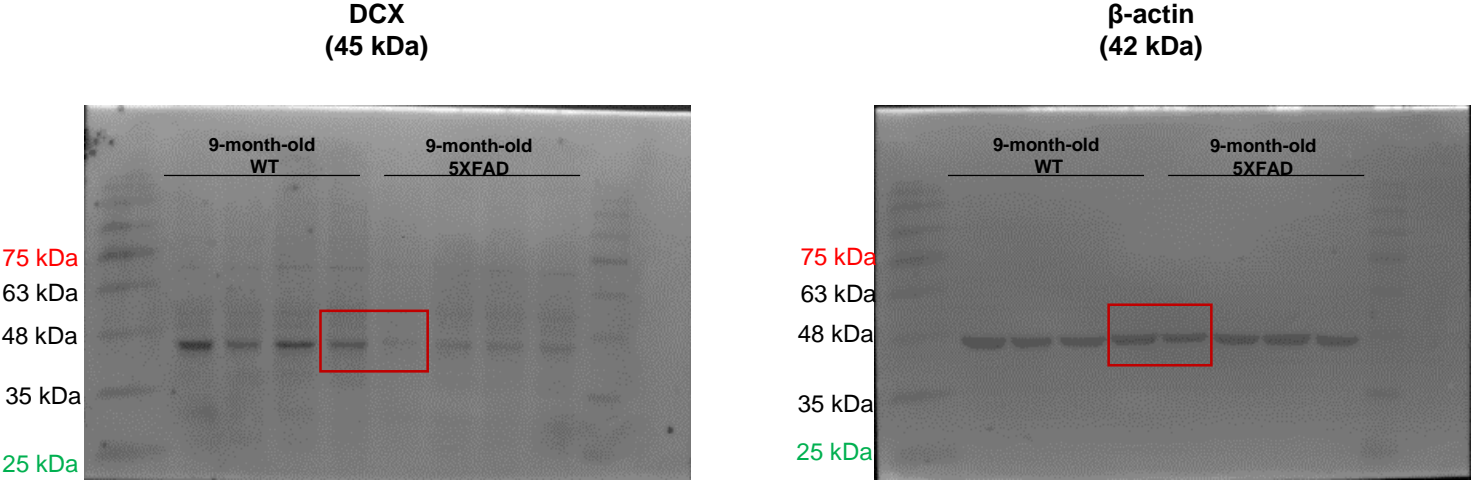

Supplement: Supplementary file 2 — Appendix S1 [file CNS-30-e14509-s002.pdf]
